# Acute and chronic effects of inspiratory muscle training in patients with type 2 diabetes mellitus: a systematic review of randomized controlled trials

**DOI:** 10.3389/fspor.2024.1423308

**Published:** 2024-12-11

**Authors:** Zoé Breuil-Marsal, Clémence Godek, Amandine Lotti, Patrick Feiereisen, Isabela Roque Marçal, Patricia Rehder-Santos, Juliana Cristina Milan-Mattos, Raphael Martins de Abreu

**Affiliations:** ^1^ Department of Health, LUNEX University of Applied Sciences, Differdange, Luxembourg; ^2^ Department of Cardiology, Centre Hospitalier de Luxembourg, Luxembourg, Luxembourg; ^3^ Exercise Physiology and Cardiovascular Health Lab, Division of Cardiac Prevention and Rehabilitation, University of Ottawa Heart Institute, Ottawa, ON, Canada; ^4^ Dr. Washington Antônio de Barros Teaching Hospital (HU UNIVASF), Brazilian Hospital Services Company (EBSERH), Petrolina, Brazil; ^5^ Department of Physical Therapy, Federal University of São Carlos, São Paulo, Brazil; ^6^ Department of Health, LUNEX ASBL Luxembourg Health & Sport Sciences Research Institute, Differdange, Luxembourg

**Keywords:** glucose, respiratory muscle training, autonomic nervous system, exercise capacity, hemodynamic

## Abstract

**Objectives:**

To conduct a systematic review to determine the acute and chronic effects of inspiratory muscle training (IMT) in type 2 diabetes mellitus (T2DM) patients on cardiac autonomic function, glucose variability, inspiratory muscle strength and endurance, hemodynamic variables, and exercise capacity.

**Methods:**

A search was carried out according to a specific search strategy, following the PRISMA statement, and three independent reviewers have undertaken the article selection process. Searches were carried out in June 2023, on the following electronic databases: EMBASE, MEDLINE (PubMed), SCOPUS (Elsevier), and Web of Science. The methodological quality of the studies was assessed using the PEDro scale. The search was limited to English-language, randomized controlled trials (RCTs), involving T2DM patients (>18 years old, with or without autonomic neuropathy, and/or inspiratory muscle weakness) following an acute or chronic intervention protocol based on IMT. Exclusion criteria were reviews, clinical trials, case studies, theses, dissertations, scientific conference abstracts, subjects with other chronic respiratory/neurological/cardiovascular diseases, and studies addressing other breathing exercises.

**Results:**

The search strategy identified 1,352 studies, of which eight (two involving acute and six involving chronic IMT effects) were included. A total of 214 adults aged 52–63 years (51/49 male/female ratio), with BMI ranging from 27 to 36.8 kg/m², were included. The results demonstrated that after IMT, acute effects were reported, such as reduced glucose levels and an increase in the parasympathetic pathway, but also chronic effects including improved inspiratory muscle strength, endurance, and exercise capacity.

**Conclusion:**

Although some methodological differences among the studies were found, IMT may have beneficial effects on cardiac autonomic function, glucose level control, inspiratory muscle strength/endurance as well as exercise capacity. However, further studies are necessary to confirm these benefits.

## 1 Introduction

Type 2 diabetes mellitus (T2DM) is a chronic metabolic disease characterized by hyperglycemia caused by insulin resistance. This resistance results from a gradual decline in sufficient β-cell insulin secretion, potentially linked to environmental or genetic factors (1–3). The International Diabetes Federation projected that the diabetes prevalence, will increase from 10.5% in 2021, to 11.3% by 2030 and 12.2% by 2040 (4). It is known that impaired muscle metabolism associated with phrenic nerve neuropathy in T2DM patients, contributes to decreased respiratory muscle strength, leading to respiratory muscle dysfunction. Although the physiological mechanisms are not fully understood, T2DM patients have shown reduced endurance in their respiratory muscles, and this decline is linked to the level of metabolic control, as measured by HbA1c levels (2, 5–8). In addition, chronic hyperglycemia leads to chronic complications such as autonomic cardiovascular neuropathy, which can affect the cardiac autonomic nervous system (1, 9, 10). A prior meta-analysis revealed diminished sympathetic and parasympathetic modulation in T2DM patients, likely due attributed to the deleterious metabolic consequences of altered glucose metabolism on cardiac autonomic regulation (9).

Contemporary literature recommends lifestyle modification, including physical activity and adherence to a well-balanced diet, as beneficial strategies for managing T2DM (11). For this population, both moderate-intensity continuous training and high-intensity interval training are recommended, with no evidence of superiority between them, and aerobic and resistance exercises are often advised (2, 12, 13). Several studies suggest that physical exercise positively influences various physiological and psychological factors in people with T2DM, including insulin sensitivity, skeletal muscle oxidative capacity, inflammation, blood glucose levels, stress, anxiety, depression, pain, mood, self-efficacy, cognitive function, cardiovascular health, and circadian rhythms (2, 14, 15). These findings indicate that physical exercise may benefit patients with T2DM by potentially enhancing their overall well-being and quality of life (15). Physical activities and dietary interventions are generally not associated with serious adverse effects. However, physical activity can sometimes lead to orthopedic injuries, and individuals with low physical fitness may face cardiovascular-related adverse effects during exercise, such as sudden increases in cardiovascular variables and, in rare cases, death. Notably, due to the low incidence of serious adverse effects, this aspect has been minimally explored in scientific studies (16).

Inspiratory muscle training (IMT) has been proposed as a complementary therapy for the management of T2DM. This type of ventilatory training has shown to decrease blood glucose levels, decrease insulin resistance and diaphragmatic breathing capacity, and increase respiratory muscle strength. It can also be used as an initial strategy prior to initiation of physical activity (17, 18). IMT has demonstrated positive effects on cardiac autonomic function, which is frequently impaired in T2DM patients, thereby increasing cardiovascular risk. IMT promotes a reduction of cardiac sympathetic modulation coupled with an elevation in cardiac parasympathetic modulation at rest (19, 20).

A recent systematic review assessed the chronic effects of IMT in T2DM, with inconclusive findings on functional capacity, glycemic control, and cardiac autonomic regulation (21). Our study, however, investigates the acute effects of IMT, focusing on immediate physiological responses that were not addressed in prior research. Additionally, we investigate previously unexplored outcomes, such as hemodynamic variables including heart rate and blood pressure. By examining these acute effects, our study aims to provide new insights into the potential mechanisms of action of IMT and its immediate impact on type 2 diabetes management.

These insights may enhance our understanding of the intervention's overall effectiveness and safety both during and immediately following its application. Therefore, this systematic review aims to assess the acute and chronic effects of IMT in T2DM focusing on cardiac autonomic function, glucose levels, inspiratory muscle strength and endurance, exercise capacity, functional capacity, and hemodynamic variables. This knowledge will deepen our understanding of IMT adaptations and adjustments in individuals with T2DM, providing valuable guidance to rehabilitation professionals. It will support the development of more effective IMT prescriptions in clinical settings, tailored to both short- and long-term treatment objectives.

## 2 Methods

This study was conducted following the Preferred Items for Systematic Reviews and Meta-analysis (PRISMA) 2020 guidelines (22), and was registered on the International Prospective Register of Systematic Reviews (PROSPERO) under the identification number: CRD42023481340.

### 2.1 Search strategy

The search was performed in June 2023, in the following electronic databases: EMBASE, MEDLINE (PubMed), SCOPUS (Elsevier), and Web of Science. It was restricted to randomized clinical trials and the English language. The following search strategy incorporated Medical Subject Headings (MeSH) with keywords was applied: (breathing exercises OR exercise, breathing OR respiratory muscle training OR muscle training, respiratory OR training, respiratory muscle OR inspiratory muscle training) AND (exercise tolerance OR exercise capacity OR glycemic control OR control, glycemic OR blood glucose control OR control, blood glucose OR glucose control, blood OR blood glucose OR hyperglycemia OR hypoglycemia OR glycemic index OR heart rate OR heart rate OR blood pressure OR respiratory muscle strength OR inspiratory muscle strength) AND (diabetes mellitus, type 2 OR diabetes mellitus).

Articles from each database were retrieved and imported into the Rayyan platform (available in: https://www.rayyan.ai). The study selection process involved three independent reviewers. Initially, two independent reviewers (A.L. and Z.B.M.) screened the titles and abstracts of all records identified following our eligibility criteria. Subsequently, full texts were screened and reviewed in order to determine inclusion in this systematic review. If there were some disagreements in some of the steps between these two independent reviewers, a third independent reviewer (C.G.) was consulted. Additionally, the reference lists of the included articles were screened to identify other possible eligible trials.

### 2.2 Eligibility and exclusion criteria

To formulate the study question and set eligibility criteria, the Population, Intervention, Comparison, Outcome, Study Design (PICOS) system was used. Eligible studies included: adult T2DM patients (P); performed an acute (<4 weeks) or chronic (≥4 weeks) IMT program (I); compared with a control group either without intervention or with a sham intervention (C); assessed cardiac autonomic function, hemodynamic variables *(*i.e., blood pressure, heart rate), glucose measurements, inspiratory muscle strength and endurance, exercise capacity, and/or functional capacity (O); were randomized clinical trials (RCTs) (S) evaluating the impact of IMT on T2DM subjects. Eligibility of selected articles was based on assessment of one or more of the following outcomes measures: cardiovascular autonomic function, inspiratory muscle strength (MIP and S-Index), glucose measurements (HbA1c or CGMS), and exercise capacity (VO2peak, VO2max or VCO2max). Eligible IMT protocols involve the use of inspiratory muscle trainer devices and the inspiratory load during the protocol must be ≥30% of the maximal inspiratory pressure (MIP) in the intervention group, as previous studies have shown that intensities lower than 30% do not promote clinically significant benefits in patients with T2DM (21). Exclusion criteria were included studies employing different types of respiratory exercises (e.g., expiratory muscle training, slow breathing) or combining other interventions during IMT protocol as well as additional comorbidities (e.g., neurological, chronic respiratory, cardiovascular, or musculoskeletal diseases).

### 2.3 Data extraction

From each selected study, data were reported through a descriptive analysis and extracted independently by two reviewers (A.L. and Z.B.M.), including: (1) characteristics of the samples, participants, and groups (sample size, group number, sex, age, MIP, T2DM diagnosis, glucose level, glycated hemoglobin (HbA1c), medication and other comorbidities) summarized in Table 1; (2) characteristics of interventions [duration, devices used, frequency, intensity, duration, respiratory frequency, intervention details, supervision, and control group (CG) intervention] reported in Table 2; and (3) outcomes description (autonomic function, glucose measurements, inspiratory muscle strength/endurance, and exercise capacity) detailed in Table 3.

**Table 1:** Study characteristics.

Study	Group (*n*)	Age (years)	Sex (M/F)	BMI (kg/m^2^)	MIP (cmH2O) baseline	T2DM diagnosis (years)	Medication	Glucose level (mg.dl-1)	Hb1Ac (%)	Other comorbidities
Ahmad and Ali (23)	IG (*n* = 12) CG (*n* = 14)	IG (42.2 ± 6) CG (44.3 ± 7)	(0/28)	IG (34.6 ± 4.6) CG (36.8 ± 5.7)	NR	IG (4 ± 3) CG (2.9 ± 3.4)	Oral hypoglycemic	IG (134.9 ± 37.3) CG (145.3 ± 46.7)	IG (6.65 ± 1.16) CG (6.75 ± 1.21)	Not mentioned
Albarrati et al. (24)	IG (*n* = 15) CG (*n* = 15)	IG (52 ± 5) CG (54 ± 3)	(20/10)	IG (29.85 ± 4.53) CG (29.46 ± 4.46)	IG (76.33 ± 9.5) CG (70.10 ± 7.7)	>5	Not mentioned	Not mentioned	Not mentioned	IMW
Correa et al. (25)	IG (*n* = 10) IG-CAN (*n* = 10) CG (*n* = 10)	IG (59 ± 8) IG-CAN (59 ± 9) CG (56 ± 7)	IG (4/6) IG-CAN (4/6) CG (6/4)	IG (26 ± 7) IG-CAN (27 ± 1) CG (25 ± 3)	IG (76 ± 17) IG-CAN (80 ± 16) CG (111 ± 13)	IG (8 (6–12) IG-CAN (11 (6–15) CG (not mentioned)	Metformin Sulfonylureas ACE inhibitor Diuretics Statins β-blockers	IG (144 ± 21) IG-CAM (141 ± 26) CG (89 ± 6)	IG (7 (7–9)) IG-CAM (8 (8–9)) CG (5 (5–6))	Autonomic neuropathy (*n* = 10)
Correa et al. (26)	IG (*n* = 12) CG (*n* = 13)	IG (63 ± 7) CG (63 ± 7)	IG (7/5) CG (5/8)	IG (27.3 ± 3.2) CG (28.2 ± 2.6)	IG (56 ± 13) CG (52 ± 10)	IG (11.6 ± 4.7) CG (13.9 ± 8.3)	Insulin Metformin Sulfonylureas ACE inhibitor Diuretics Statins β-blockers	IG (152 ± 49) CG (134 ± 72)	IG (7.5 ± 1.4) CG (7.1 ± 1.7)	IMW
Kaminski et al. (19)	IG (*n* = 5) CG (*n* = 5)	IG (56 ± 9) CG (55 ± 10)	Not mentioned	Not mentioned	IG (88 ± 26) CG (98 ± 34)	IG (13 ± 1) CG (10.7 ± 6)	Not mentioned	Not mentioned	Not mentioned	Not mentioned
Moawd et al. (27)	IG (*n* = 28) CG (*n* = 27)	IG (55.5 ± 9.8) CG (59.5 ± 4.8)	IG (20/8) CG (22/5)	IG (29.2 ± 3.9) CG (27.9 ± 4.8)	IG (56 ± 13) CG (52 ± 10)	Not mentioned	Not mentioned	Not mentioned	IG (7.3 ± 1.6) CG (7.2 ± 1.9)	Peripheral neuropathy with obstructive sleep apnea IMW
Pinto et al. (28)	IG (*n* = 11) CG (*n* = 13)	IG (59.5 ± 9.2) CG (59.6 ± 12.3)	IG (4/7) CG (4/9)	IG (28.5 ± 3.2) CG (27.0 ± 3.1)	IG (89.2 ± 5.1) CG (100 ± 5.1)	IG (10 (5 -15)) CG [9 (6 -12)]	Insulin Metformin Sulfonylureas ACE inhibitor Diuretics Statins β-blockers Anticoagulant AR blocker	IG (190.2 ± 16) CG (167.1 ± 16.1)	IG (8.6 ± 0.2) CG (8.8 ± 0.2)	Autonomic neuropathy [IG (3); CG (2)]
Shein et al. (29)	(*n* = 14)	IG (53.6 ± 7.4) CG (53.6 ± 7.4)	(8/6)	(29.6 ± 3.7)	(94.1 ± 38.8)	[6.5 (2.7–11)]	Metformin Sulfonylureas Other antidiabetic drugs Ca^2^^+^ channel blockers AR blocker ACE inhibitor Diuretics Statins β-blockers Antiplatelet	(196.8 ± 35.8)	(8.8 ± 0.9)	Autonomic neuropathy (*n* = 1)

M, Male; F, Female; BMI, body mass index; MIP, maximal inspiratory pressure; HbA1c, glycated hemoglobin; IG, intervention group; CG, control/sham group; CAN, cardiovascular autonomic neuropathy; IMW, inspiratory muscle weakness (MIP <70%); ACE inhibitor = angiotensin-converting enzyme inhibitor; AR blocker, angiotensin II receptor blocker; Ca^2+^ channel blocker, calcium channel blocker; NR, not reported.

**Table 2:** Intervention description.

Study	Duration (weeks)	Device	Frequency (days/week)	IMT duration per session (min)	IG intensity (% MIP)	CG	Respiratory frequency	Intervention details	Supervision
Ahmad and Ali (23)	8 weeks	Threshold IMT	5 days/week	Weeks 1–2: 10–15 min Weeks 3–5: 15–20 min Weeks 6–8: 20–25 min	30%	Medication only	5 sets × 20 breaths/set 5 sets × 30 breaths/set 8 sets × 30 breaths/set	Deep and slow DB	Supervised by a physiotherapist
Albarrati et al. (24)	8 weeks	Threshold IMT	7 days/week	30 min	40%	Sham: 15% of MIP	15–20 breaths/min	DB Weekly MIP assessment Incremental protocol	1x week supervised session 6x/week non-supervised session
Correa et al. (25)	Single session	IG + IG-CAN (POWERbreathe®) CG (Threshold IMT)	1 day/week	13 min	60%	Sham: 2% of MIP	15 breaths/min	Spontaneous breathing (5 min) And controlled breathing (3 min) with audio feedback	One supervised session
Correa et al. (26)	8 weeks	Threshold IMT	7 days/week	30 min	30%	Sham: Lowest pressure (7 cm H2O)	Not mentioned	DB Weekly MIP assessment	1x week supervised session 6x/week non-supervised session
Kaminski et al. (19)	8 weeks	Threshold IMT	7 days/week	30 min	30%	Sham: No load	15–20 breaths/min	DB	Not mentioned
Moawd et al. (27)	12 weeks	TRAINAIR®	3 days/week	30 min	75%	Sham: ≤10% of MIP	6 sets of 30 breaths	Biofeedback	Supervised by a professional physiotherapist
Pinto et al. (28)	12 weeks	POWERbreathe®	7 days/week	30 min	30%	Sham: Minimal resistance (2% of MIP)	15–20 breaths/min	DB Weekly MIP assessment	1x week supervised session 6x/week non-supervised session
Shein et al. (29)	Single session	IG (POWERbreathe®) CG (Threshold IMT)	1 day/week	70 min	60%	Sham: Minimal resistance (2% of MIP)	15 breaths/min	Day 1: CGMS Day 2: Intervention (a) Controlled ventilation (b) IMT Day:3: glucose sensor removal Audio and visual feedback	Supervised session

IMT, inspiratory muscle training; IG, intervention group; CG, control/sham group; CAN, cardiovascular autonomic neuropathy; DB, diaphragmatic breathing; OM, outcome measure; CGMS, continuous glucose monitoring system.

**Table 3:** Main outcomes.

Study	Autonomic function	Glucose measurement	Inspiratory muscle strength/endurance	Exercise capacity	Hemodynamic variables
Ahmad and Ali (23)	NA	No statistical difference	Not assessed	NA	NA
Albarrati et al. (24)	NA	NA	Increased MIP	Increase in 6MWT, and TUG scores	
Correa et al. (25)	NA	Glucose level reduction in 6 participants (IG-CAN = 4; IG = 2) during IMT at 60% of MIP	Not assessed	Not assessed	HR and CBF reduction in IG-CAN. FVR and MAP during exercise were higher in both groups
Correa et al. (26)	No statistical difference	NA	Increased MIP Pthmax/MIP and endurance time increase.	No statistical difference	NA
Kaminski et al. (19)	Reduced sympathetic cardiac modulation (LFnu)	NA	Significant increased MIP after 8 weeks	No statistical difference	NA
Moawd et al. (27)	NA	NA	Increased MIP	VO2max and VCO2max improvement	NA
Pinto et al. (28)	NA	Reduction in fasting plasma glucose levels at 30% MIP after 8 weeks with no changes after 12 weeks. No differences in HbA1c levels.	PTI increase.	Not assessed	NA
Shein et al. (29)	No statistical difference	No statistical difference	NA	NA	Higher HR as compared to SHAM load, at the 1st min; higher MAP and HR at the 2nd min and also in the last minute of IMT

MIP, maximal inspiratory pressure; NA, not assessed; 6MWT, six-minute walk test; FVR, forearm vascular resistance; TUG, timed up and go; HR, heart rate; CBF, calf blood flow; IG-CAN, intervention group with cardiovascular autonomic neuropathy; CVR, calf vascular resistance; MAP, mean arterial pressure; IG, intervention group; IMT, inspiratory muscle training; Pthmax, inspiratory muscle endurance; LFn, normalized unit of low frequency power; PTI, pressure time index; VO2max, maximal oxygen consumption; VCO2max, maximal carbon dioxide exhaled; HbA1c, glycated hemoglobin.

### 2.4 Quality assessment

The PEDro quality scale for RCTs was used to assess the quality of the included studies. This scale comprises 11 items including eligibility criteria, randomized allocation, concealed allocation, comparable at baseline, blinded subjects, blinded therapists, blinded assessors, adequate follow-up, intention to treat analysis, between-group comparisons, point estimates and variability. The first item does not contribute to the score, resulting in a maximum achievable score of 10 points. Moreover, the methodological quality of articles was categorized as poor (<4 points), fair (4–5 points), good (6–8), or excellent (9–10), based on previous quality criteria (30). Quality assessment was conducted independently by two reviewers (A.L. and Z.B.M.) and in case of disagreements between them, a third reviewer (C.G.) was consulted.

## 3 Results

### 3.1 Study selection

**Figure 1:**
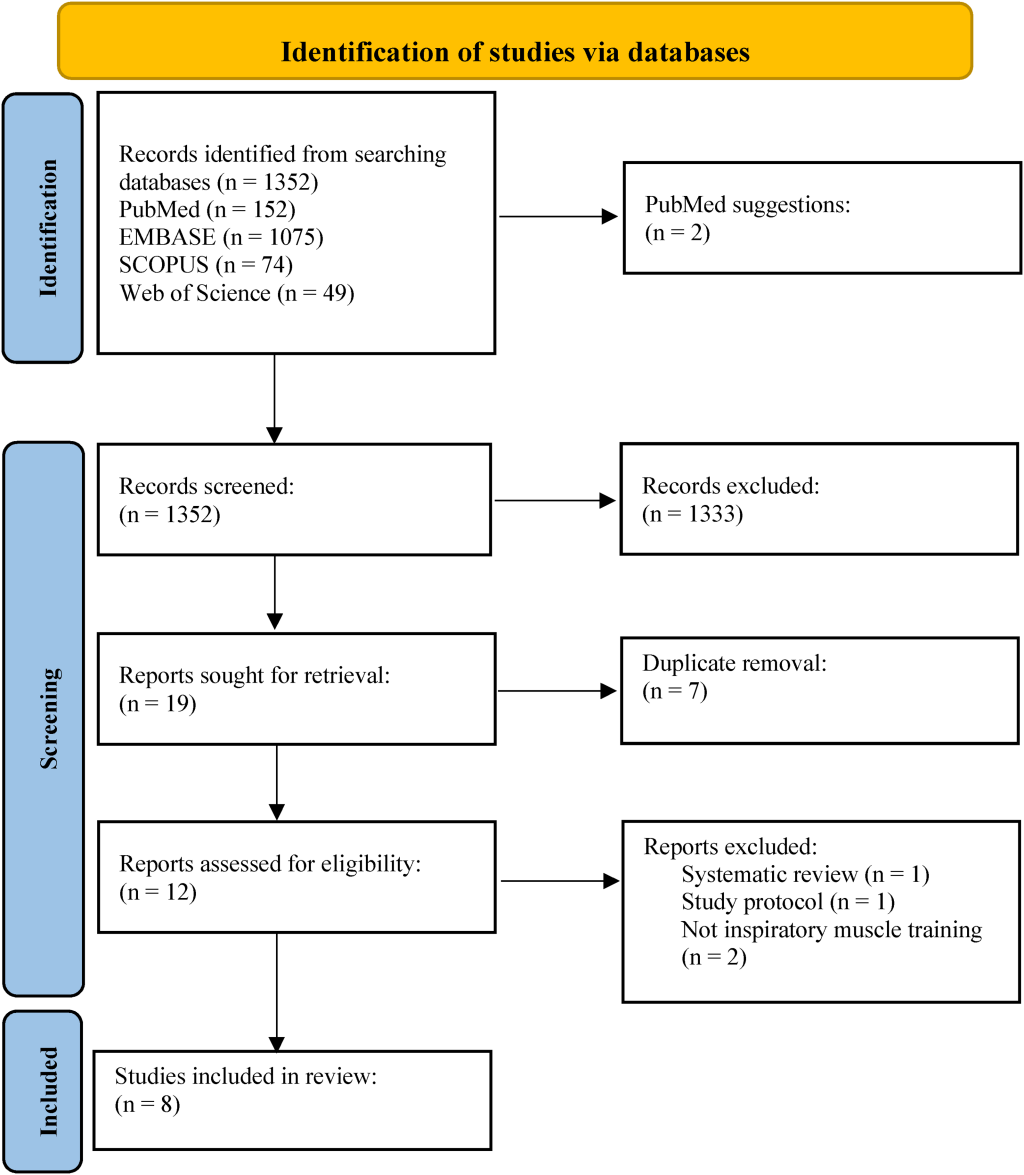
PRISMA flowchart.

A PRISMA ﬂow diagram of the literature search and selection is presented in Figure 1. The initial search identified a total of 1,352 potential studies through the database search (EMBASE = 1,075, PubMed = 152, SCOPUS = 74, Web of Science = 49) and PubMed additional studies suggestions. A total of 342 duplicates were identified and removed. From the titles and abstract reading, 17 studies were selected for full-text reading. Out of 17 studies, 11 were excluded, six were included and two studies retrieved from PubMed suggestions were screened and accepted for inclusion (Figure 1). As a result, eight studies were included in this systematic review.

### 3.2 Participant characteristics

A total of 214 adults aged 52–63 years were included, with male (*n* = 104) and female (*n* = 100) proportions of 50.9% and 49.1%, respectively. One study did not report the sex of the participants (19). Seven studies included participants diagnosed with T2DM with more than five years of diagnosis (19, 24–29). According to the body mass index (BMI) classification, one study included Class I obese participants in the intervention group (23), and seven studies included overweight subjects (19, 24–29). Some participants were also presented with autonomic neuropathy or inspiratory muscle weakness, when their MIP were inferior to 70% of predicted value. Additionally, four studies have included some patients under beta-blockers (25, 26, 28, 29). More details of the study characteristics are reported in Table 1.

### 3.3 Intervention characteristics

Different protocols including frequency, intensity, time, and type (FITT principles) were used to evaluate both acute (single session) and chronic effects (ranging from 8 to 12 weeks). The interventions ranged from 13 to 70 min, occurring 3–7 days per week. Two studies focused on acute effects, delivering IMT in a single session (25, 29), with an intensity of 60% of MIP in the intervention group (25, 29). Chronic IMT effects were investigated in six studies, with intervention durations ranging from 8 to 12 weeks (19, 23–25, 27, 28).

In these studies, the commonly applied training intensity ranged from 30% to 40% of MIP, as implemented in five of the included studies (19, 23, 24, 26, 28). Conversely, only one study applied a higher IMT intensity at 75% of MIP (27). The frequency of training ranged from 3 to 7 days a week (19, 23, 24, 26–28). Moreover, seven studies included at least one supervised session with a physiotherapist (23–29). Considering the IMT devices, seven studies utilized either a POWERbreathe® or a Threshold IMT device (19, 23–26, 28, 29), while one study used a TrainAir device with biofeedback (27).

Of the 8 studies included in this review, 7 of them compared the intervention group to the sham group, based on the same protocol but with a training intensity ranging from no resistance (0% of MIP) up to 15% of MIP. Finally, one study compared the IMT effects to the usual care, based on medication only (23). For more comprehensive details, please refer to Table 2.

### 3.4 Outcomes

The outcomes reported in this systematic review were summarized in Table 3, including: (1) heart rate (HR), heart rate variability (HRV), mean arterial pressure (MAP), blood pressure (BP), forearm vascular resistance (FVR), calf blood flow (CBF), and calf vascular resistance (CVR); (2) glucose measurement through plasma glucose and glycated hemoglobin (HbA1c); (3) inspiratory muscle strength determined by the MIP, the Sniff nasal inspiratory pressure (SNIP) and inspiratory muscle endurance represented by the ratio of the maximum threshold pressure (Pthmax) to MIP; (4) exercise capacity and/or functional capacity assessed either by cardiopulmonary exercise test (CPET), 6-minutes walking test (6MWT) or Timed Up and Go (TUG).

#### 3.4.1 Cardiac autonomic modulation

Cardiac autonomic modulation was evaluated in four studies (19, 25, 26, 29). One study concluded that IMT had no significant effect on HRV in time and frequency domains (26). A study reported a decrease in the power spectrum of the high-frequency band in normalized units (HF_nu_) (29). Another study proposed that IMT contributed to the reduction in sympathetic cardiac modulation by decreasing the normalized unit of the low-frequency (LF_nu_) power component of HRV (19). Regarding HR, three studies underwent analysis, each yielding conflicting data (25, 27, 29). One study observed lower HR at the end of the protocol in the T2DM and cardiovascular autonomic neuropathy (T2DM-CAN) group compared to the T2DM and control groups (25). One study showed a non-significant decrease in HR in the intervention group compared to the control group (27). One study revealed that the HR was higher in the experimental load compared to the sham load during the first, second, and last minute of intervention (29). Two studies showed an increase in MAP during the training session (25, 29). Two studies evaluated the BP, one highlighted a non-significant reduction in systolic and diastolic BP at rest (27), and another study showed an increase in BP during the protocol (29). Only one study investigated CBF and CVR, exposing a reduction in CBF and an increase in CVR in the T2DM and T2DM-CAN during the intervention in experimental groups compared with the control group (25).

#### 3.4.2 Glucose levels

Four studies evaluated glucose levels (23, 25, 28, 29). Two studies evaluated fasting plasma glucose levels (23, 28). In one study, there was a 106% reduction after the protocol (28), while the second study, although lacking statistically significant outcomes, revealed glucose values below 130 mg/dl, suggesting clinical significance (23). One study found no changes in HbA1c levels (28). One study showed a reduction in glucose levels over time without changes in glucose variability (29). One study illustrated a reduction in glucose levels evaluated by a continuous glucose monitoring system (CGMS) (25).

#### 3.4.3 Inspiratory muscle strength and endurance

The assessment of inspiratory muscle strength was carried out in five distinct studies (19, 24, 26–28). Four studies demonstrated an improvement in inspiratory muscle strength (19, 24, 26, 27). One study reported no changes in inspiratory muscle strength (28). Additionally, two studies appraised muscle endurance, revealing an increase in endurance time and in the pressure time-index (PTI) characterizing maximal threshold pressure divided by MIP (26, 28).

#### 3.4.4 Exercise capacity

Four studies evaluated exercise capacity (19, 24, 26, 27). One study employed the 6MWT, TUG, and HGS, demonstrating improvement in all three functional tests within both groups from baseline to the end of the protocol. The experimental group showed greater improvement than the sham group (24). Three studies used CPET with either a treadmill (26, 27) or a cycle ergometer (19). One study identified improvement in aerobic capacity between groups, with increased VO_2_max to IMT (27). Two studies reported no significant changes in exercise capacity (19, 26).

### 3.5 Quality appraisal

The items, total score of the PEDro scale and the quality evidence were described in Table 4. The highly variable scores (between 4 and 9) highlighted the heterogeneous methodological quality of the studies, with an average score of 5.5 points, which is considered “fair”. Five studies were classified as fair (19, 23, 25, 27, 29), two studies as good (24, 26) and one study as excellent (28) according to the Cashin & McAuley classification (30). The main items not reported in the included studies were unconcealed group allocation and blinding of subjects, therapists, and assessors. Although it would be important to consider these factors in future studies, it is possible that blinding process may have been limited by the study design and type of intervention adopted.

**Table 4:** PEDRO scale.

Study	1	2	3	4	5	6	7	8	9	10	11	Total	Quality of evidence
Ahmad and Ali (23)	Yes	0	0	1	0	0	0	1	0	1	1	4	Fair
Albarrati et al. (24)	Yes	1	1	1	0	0	0	1	1	1	1	7	High
Correa et al. (25)	Yes	1	0	1	0	0	0	0	0	1	1	4	Fair
Correa et al. (26)	Yes	1	0	1	1	0	1	0	0	1	1	6	High
Kaminski et al. (19)	Yes	1	0	1	0	0	0	0	0	1	1	4	Fair
Moawd et al. (27)	Yes	1	0	1	0	0	0	1	0	1	1	5	Fair
Pinto et al. (28)	Yes	1	1	1	1	0	1	1	1	1	1	9	High
Shein et al. (29)	Yes	1	0	1	0	0	0	1	0	1	1	5	Fair

Criteria: (1) Eligibility; (2) Random allocation; (3) Concealed allocation; (4) Similar groups at baseline; (5) Blinding of subjects; (6) Blinding of therapists; (7) Blinding of assessors; (8) Patients’ follow-up; (9) Intention to treat analysis; (10) Comparison between groups; (11) Point measures and variability.

## 4 Discussion

This systematic review aimed to determine the acute and chronic effects of IMT on different aspects of patients with T2DM. After careful analysis of the eight RCTs included in our study, with a total sample size of 214 participants, IMT demonstrated beneficial effects both in the short and long term. Of these studies, two focused on acute effects and assessed the cardiac autonomic modulation and glucose levels (25, 29), while six assessed chronic effects by evaluating inspiratory muscle endurance and strength, and exercise capacity. It is known that T2DM affects the whole body, including the cardiovascular, muscular, and/or respiratory systems (2, 5, 6, 8). Several studies have demonstrated increased insulin resistance, neuromuscular alterations and poor blood perfusion (1, 24) causing inspiratory muscle weakness (19), reduced exercise capacity (24), and alterations of autonomous function. IMT may delay or counter inspiratory muscle weakness and improve inspiratory muscle strength (24). Similarly to obese patients, IMT led to an improvement in inspiratory muscle strength and exercise capacity (31). According to the results reported in this systematic review, an increased hemodynamic response was observed during IMT however these changes were not sustained after session. IMT may promote acute decease in the resting cardiac sympathetic modulation and a reduction in glucose levels although only one study assessed these effects. In the long-term effects, inspiratory muscle strength and endurance were increased, also resulting in increased exercise capacity. Consequently, the use of IMT appears to be a non-conventional training option for T2DM patients. Furthermore, IMT may serve as a supplementary approach, particularly beneficial for individuals who are not compliant with traditional rehabilitation methods (32–34).

### 4.1 Acute IMT effects

Acutely, IMT at intensities of 60% of MIP caused exaggerated peripheral vasoconstriction in T2DM, suggesting hyperactivation of the inspiratory metaboreflex. This activation can lead to higher HR and MAP, as well as reduced parasympathetic cardiac activity, as observed during the experimental session at 60% of MIP, but not with the sham load (25, 29). It is well known that inspiratory metaboreflex activation during strenuous exercise primarily increases MAP by enhancing cardiac output. However, these changes on hemodynamic variables were not sustained after the exercise ceased. Additionally, glucose levels significantly decreased during inspiratory muscle metaboreflex activity and remained low for at least 30 min after the acute session. The abrupt decrease in glucose levels observed during inspiratory loading was of a similar magnitude to the acute reduction in glucose levels observed after acute aerobic exercise in a similar population. This is consistent with subsequent findings of increased GLUT4 protein content in the sheep diaphragm induced by chronic inspiratory resistive flow. Controversially, although a reduction in glucose was observed 5 min and 120 min after the IMT session, no differences were found between the inspiratory loads (i.e., 60% of MIP vs. sham group). One of hypothesis is that controlled ventilation, applied before both experimental loads (2% and 60% of PImax), would be the determinant of glucose reduction (29). Controlled ventilation induces vagal stimulation, resulting in insulin secretion and glucose reduction. This is consistent with studies indicating that parasympathetic activation decreases hepatic glucose release and enhances insulin secretion in hyperglycemic conditions (35). Additionally, afferent vagal nerve stimulation leads to a sustained increase in glucose levels, partly mediated by the suppression of pancreatic insulin secretion. Conversely, efferent vagal nerve stimulation prompts pancreatic glucagon secretion, which seems to be counteracted by insulin secretion during selective efferent vagal nerve stimulation (36, 37).

### 4.2 Cardiac autonomic modulation

It is known that inspiratory muscle weakness (IMW) and cardiovascular autonomic neuropathy (CAN) induced by T2DM can lead to resting sympathetic hyperactivity (20) related to an impairment of the cardiovascular autonomous control, as result an increased resting HR are often observed in these patients (38). When non-invasively assessed by HRV, Corrêa et al. (26) reported no significant difference in HRV despite employing a comparable protocol to Kaminski et al. (19), who found a positive reduction in sympathetic modulation indicated by decreased resting LFnu indexes after IMT, provides valuable insights into the effects of IMT. Although the mechanisms influencing HRV after IMT are conflicting in the literature (20), IMT may provide beneficial effects on the autonomic nervous system by increasing the resting parasympathetic activity and/or reducing the sympathetic activation, which is considered a cardioprotective response (20, 39–41). The physiological mechanisms involved in these changes might be related to improvements on baroreflex and metaboreflex sensitivity (42).

It is known that slow breathing and diaphragmatic breathing have been associated with increased baroreflex sensitivity in healthy and pathological conditions which might contribute for an improved cardiovascular autonomic regulation (20, 43, 44), however, greater effects on baroreflex have been observed when an inspiratory load is applied (44, 45). Furthermore, an increase in inspiratory muscle strength following loaded IMT is associated with a delay in the metaboreflex threshold, a reflex mediated by peripheral sympathetic activity. Both baroreflex and metaboreflex are physiological reflexes that respond to metabolic changes. The arterial baroreflex plays an essential role in BP adjustments given the baroreceptors in the carotid and aortic arteries (46). Alterations in BP due to changes in intrathoracic pressures during IMT, might lead to the stretching of these baroreceptors due to changes in the venous return, improving the baroreceptors responses to afferent neuronal firing driven by respiration (46). Consequently, this improved baroreflex sensitivity after chronic IMT, might promote a better autonomic balance, increasing the parasympathetic nerve activity and decreasing the sympathetic nerve activity at rest (46). While the intake of beta-blockers predominantly affects the autonomic nervous system by antagonizing beta-adrenergic receptors, which are pivotal in sympathetic nervous system function, it is noteworthy that only a minority of patients in the sample were taking beta-blocker medications.

Numerous studies have demonstrated the efficacy of IMT in lowering BP, achieved through the inhibition of sympathetic and the stimulation of parasympathetic modulation (43). Slow breathing and diaphragmatic breathing have proven to be a key contributor to BP reduction (43, 44). Furthermore, the application of resistance as during IMT has been proved to intensify negative intrathoracic pressures during the training, enhancing the impact on baroreflex sensitivity due to changes on venous return, and amplifying the reduction in BP (47). In one study, an elevation in BP, HR, and reduction of the HF_nu_ component was noted, however, it is essential to contextualize that in this study, the outcome measures were assessed during the intervention (29). This finding aligns with expectations, as the heightened cardiovascular demands and increased oxygen requirements in active muscles during exercise typically increase sympathetic activity, leading to elevated HR and BP (48). Also, during exercise, the parasympathetic modulation is expected to decrease explaining the HF_nu_ reduction (49). However, contrasting findings suggested that IMT did not impact the baroreflex in non-clinical populations and younger individuals (42, 50). Suggesting that more research is needed on the effects of IMT on baroreflex in subjects with T2DM.

### 4.3 Glucose levels

Regarding glucose assessment, post IMT, patients exhibited reductions in both overall glucose levels and fasting blood glucose (25, 28). The improved inspiratory muscle strength after training, which is strongly related to and delays the activation of inspiratory metaboreflex (51, 52), can decrease dyspnea levels during exercise and enhance exercise tolerance, enabling individuals with T2DM to adopt a more physically active lifestyle. We hypothesize that an increased physical activity levels after IMT is crucial for elevating skeletal muscles blood glucose consumption, given that skeletal muscles in healthy and diabetic populations eliminate approximately 85% of blood glucose (53, 54). This phenomenon is attributed to the increase in glucose clearance facilitated by insulin stimulation and muscle contraction during exercise, which results in the redistribution and increase of the glucose transporter 4 translocation (GLUT4) at the cell surface, thus improving glucose utilization (54). In four studies assessing glucose levels, only two identified effects on glucose reduction at intensities of 60% and 30% of the 1RM in acute and chronic forms, respectively (25, 28).

Moreover, a reduction in glucose levels may also be dependent on intensity levels and protocol duration. While positive benefits have been noted in cardiovascular disease patients at intensities equivalent to 60% of MIP, future researches should investigate the potential impacts of high-intensity programs (i.e., >60% of MIP), as well as prolonged protocol durations specifically in patients with T2DM (55). Higher intensities combined with longer durations of IMT may yield more effective results in reducing blood glucose levels, fasting blood glucose as well as HbA1c (56–58). However, further studies are needed to identify optimal intensities and protocol durations.

Another hypothesis that could explain this drop in glucose is the intake of medication and the level of insulin secreted during a meal. Schein et al. (29) hypothesized that the elevation of insulin levels is induced by the meal, as well as by the medication, leading to a reduction in glucose levels. Although participants in seven studies were on medication, only one study reported a change in dosage due to medical recommendation (28). The lack of data makes it impossible to conclude whether IMT is solely responsible for the change in anti-diabetic drug dosage. Further studies are also needed to understand these changes. The hypothesis of controlled ventilation would also be decisive in glucose reduction. According to Tanida et al. (35), controlled ventilation would enable vagal stimulation, leading to insulin secretion, and thus glucose reduction.

Furthermore, obstructive sleep apnea is common condition present in patients with T2DM and can impair glucose tolerance through mechanisms such as intermittent hypoxia and increased sympathetic activity, potentially worsening glucose control (59). Given that IMT can enhance respiratory muscle strength and reduce obstructive sleep apnea severity, it is plausible that IMT could offer additional benefits by improving sleep apnea, which may subsequently lead to better glucose control (60). However, only one study included in this review reported on the occurrence of obstructive sleep apnea in T2DM patients or its improvement following IMT interventions, which should be further investigated (27).

Recent systematic reviews with meta-analyses suggest that aerobic exercise (61), combined exercise (62), resistance training (63), and high-intensity interval training (64) are beneficial for reducing glycemic levels in the diabetic population. Additionally, recent topic reviews evaluating other exercise modalities, such as Pilates and Yoga, in populations with obesity/overweight, also indicate improvements in parameters such as glucose metabolism (65, 66). Therefore, the findings of this systematic review suggest that IMT could serve as an additional and complementary strategy for enhancing glucose metabolism, as it offers glycemic benefits comparable to those reported in the literature for other forms of exercise as previously mentioned.

### 4.4 Inspiratory muscle strength and endurance

Enhancements in inspiratory muscle strength reported in four studies involving long-term protocols may be explained by the presence of IMW among patients with T2DM (19, 24, 26, 27). Subjects with IMW presenting a pre-training MIP of 56 ± 13 cmH2O, improving to 121 ± 22 cmH2O post-training (26, 27), while subjects without IMW presented a pre-training MIP of 88 ± 26 cmH2O and a post-training MIP of 137 ± 27 cmH2O (19). Dall'Ago et al. (67) stated that IMT significantly improved inspiratory muscle strength in patients with chronic heart failure accompanied by IMW, and Basso-Vanelli et al. (68) reported that patients with respiratory muscle weakness (RMW) were more likely to improve their strength and endurance with IMT than those without RMW. The reduction of respiratory muscle strength in T2DM patients is mainly due to impaired respiratory neuromuscular function caused by polyneuropathy (69). However, the mechanisms behind the development of IMT in T2DM patients are still poorly understood; therefore, more studies should be conducted to understand the role of IMW development in this specific population (26). IMT may offer more pronounced benefits for people with IMW than for those without, especially as studies agree that IMT increases diaphragmatic strength, thereby delaying diaphragmatic fatigue and activation of accessory inspiratory muscles (24). As well as the patient's physical condition, the intensity of the MIP may also explain the contradictory findings within the studies analyzed in this systematic review. The MIP intensity was set at 30%, a level that might be considered insufficient, especially considering that optimal enhancements in muscle strength have been observed at 60% of the MIP for both heart failure patients, with or without IMW (55). Regarding inspiratory muscle endurance, a low intensity (30% of MIP) seems to be sufficient to achieve significant improvement, such intensity may stimulate slow-twitch fibers which are characterized by their ability to sustain contractions for extended period (26, 28). Cho et al. (70) and Britto et al. (71) confirm this hypothesis, reporting that 30% of MIP intensity showed improvement in respiratory muscle endurance in patients with stroke.

### 4.5 Exercise capacity

Two studies showed a significant improvement in exercise capacity, using an intensity superior or equal to 40% of MIP, while the other two studies found no improvement, using an intensity of 30% of MIP. One hypothesis is that engaging in moderate to high-intensity IMT confers greater advantages in enhancing inspiratory muscle strength and, subsequently, exercise capacity. This enhancement is facilitated by the delayed onset of the inspiratory metaboreflex during physical exertion (72). This activation pattern reduces blood flow to the inspiratory muscles during exercise while enhancing blood perfusion in peripheral muscles (24). Notably, in healthy individuals, IMT delays the inspiratory metaboreflex, leading to reduced sympathetic activation and diminished peripheral vasoconstriction, factors that can otherwise limit exercise capacity (73). Consequently, enhancing diaphragmatic strength through IMT has been shown to alleviate the severity of dyspnea and boost both exercise and functional capacity across diverse populations (74, 75).

### 4.6 Adverse effects

Although a limited number of studies report data on the adverse effects of IMT, it has been shown to be safe and well-tolerated in patients at risk of prolonged hospitalization, asthmatic subjects and children suffering from neuromuscular diseases (76–78). Some minor adverse effects such as muscle soreness has been reported (79). After IMT, Pinto et al. (28) reported hyperglycemia and/or hypoglycemia, fatigue, dyspnea, nausea, dizziness, and headaches, which may be uncorrelated with the application of IMT. Instead, hyperglycemia or hypoglycemia may result from dietary habits taken by the patient (80). Elevated blood glucose levels can also be explained by increased inspiratory load, as high-intensity exercise can prevent blood sugar from dropping by increasing counter-regulatory hormones, which can lead to an increase in blood glucose levels (81). The observation of increased GLUT4 protein content in the sheep diaphragm points in the same direction (82). Presence of dyspnea, for its part, may result from the patient's sedentary lifestyle but also from the respiratory muscular atrophy caused by diabetes (83).

## 5 Limitation

The present systematic review was limited by (1) the heterogeneity of intervention protocols regarding duration, frequency, device, supervision, (2) the intensity of MIP used (varying from low-intensity to high-intensity), (3) different patient characteristics such as the pathologies included (inspiratory muscle weakness or autonomic neuropathy), lifestyle (sedentary or active), gender, drug treatments, different ranges of age, and/or diet (4) the small sample size not being representative of or significant to the study population, (5) the lack of information concerning the sample calculation of certain studies representing potential attrition biases, as well as the lack of information on some dropouts. In addition, two of the included studies with a short-term protocol mainly assessed autonomic function and glucose measurement (25, 29), whereas the other six studies with a long-term protocol focused mainly on inspiratory muscle endurance and strength, and exercise capacity (19, 23, 24, 26–28). Additionally, the studies did not assess the same outcome measures for the same duration of intervention, which makes it difficult to compare the findings among them.

## 6 Conclusion

This systematic review identified eight studies assessing the acute and chronic effect of IMT in patients with T2DM. Respiratory training has demonstrated acute positive effects on autonomic function and glucose levels depending on the intensity of MIP chosen. Chronic effects such as increased strength and endurance of the inspiratory muscles, and improved exercise capacity can also be observed. There is only one study that mention the positive chronic effects on autonomic function. IMT can be presented as a complementary tool to conventional training in T2DM patients. However, the results must be interpreted with caution, as some of the included studies presented biases (e.g., selection, attrition, and intervention biases). Further studies are needed to evaluate all outcome measures (cardiac autonomic modulation, blood glucose levels, inspiratory muscle strength/endurance and exercise capacity), including a standardized short- and long-term intervention protocol and a homogeneous sample, to obtain more accurate and meaningful results.
